# Paclitaxel-Coated Balloons: Investigation of Drug Transfer in Healthy and Atherosclerotic Arteries – First Experimental Results in Rabbits at Low Inflation Pressure

**DOI:** 10.1007/s10557-016-6658-1

**Published:** 2016-04-01

**Authors:** Nicola Stolzenburg, Janni Breinl, Stephanie Bienek, Milosz Jaguszewski, Melanie Löchel, Matthias Taupitz, Ulrich Speck, Susanne Wagner, Jörg Schnorr

**Affiliations:** Department of Radiology, Section of Experimental Radiology, Charité - Universitätsmedizin Berlin, Campus Charité Mitte, Charitéplatz 1, 10117 Berlin, Germany; InnoRa GmbH, Berlin, Germany; Department of Cardiology, Charité - Universitätsmedizin Berlin, Campus Benjamin Franklin, Berlin, Germany

**Keywords:** Atherosclerotic plaque, Drug-coated balloon, Inflation pressure, Paclitaxel, Atherosclerotic rabbit model

## Abstract

**Purpose:**

Beyond antiproliferative properties, paclitaxel exhibits anti-inflammatory activity, which might be beneficial in the local treatment of nonocclusive coronary artery disease. Paclitaxel release and tissue concentrations after paclitaxel-coated balloon treatment using different pressures have not been investigated so far. The aim of the study was to investigate in an atherosclerotic rabbit model whether drug transfer from paclitaxel-coated balloons into the vessel wall is affected by the presence of atherosclerotic lesions and to which extent it depends on the inflation pressure used.

**Methods:**

Paclitaxel-coated balloons (3.5 μg/mm^2^ paclitaxel) were inflated with pressures of 1, 2, or 6 atm (60s) in healthy (*n* = 39) and atherosclerotic (*n* = 22) arteries of New Zealand White Rabbits. Paclitaxel content in arterial walls (10 min after interventions) and paclitaxel remaining on balloons after treatment were analyzed using high-performance liquid chromatography.

**Results:**

Median paclitaxel tissue concentrations were 829.3 μg/g (IQR 636.5–1487 μg/g) in healthy and 375.7 μg/g (IQR 169.8–771.6 μg/g) in atherosclerotic arteries (*p* = 0.0002). The paclitaxel tissue concentration was dependent on inflation pressure (1 atm vs. 2 atm vs. 6 atm) in atherosclerotic arteries (*p* = 0.0106) but not in healthy arteries (*p* ≥ 0.05).

**Conclusions:**

Atherosclerotic lesions impede the transfer of paclitaxel into arterial walls. Higher inflation pressures resulted in an increased paclitaxel transfer in atherosclerotic but not in healthy arteries. However, it is assumed that the tissue concentrations achieved with an inflation pressure of 2 atm are potentially effective in this model.

## Introduction

The use of drug-eluting stents (DES) has significantly reduced the reintervention rate for repeat target lesion revascularization in coronary arteries due to restenosis to below 10 % [1–4]. Following the advent of DES, drug-coated balloons (DCB) were developed as an additional option for local delivery of antiproliferative agents. Since 2009, paclitaxel-coated balloons (PCB) have been successfully used for different indications including local treatment of coronary in-stent restenosis and of stenosis and restenosis in the peripheral arteries [5–9]. Paclitaxel is an antiproliferative drug with anti-inflammatory activity [10–12]. Interestingly, the very short period of DCB inflation is sufficient to transfer enough paclitaxel into the vessel wall for long-term prevention of restenosis [13–15].

In patients with unstable atherosclerotic lesions, delivery of a high enough concentration of paclitaxel from the PCB should be ensured at low inflation pressure. Biological factors such as local inflammatory reactions or intraplaque hemorrhage and mechanical factors may result in weakening and ultimately rupture of vulnerable or unstable plaques [16, 17]. To the best of our knowledge, there are no studies that have investigated whether drug delivery into the atherosclerotic vessel wall is possible at low inflation pressure (1–6 atm). Therefore, we performed the first study to investigate the transfer of paclitaxel from PCB into atherosclerotic vessel walls in a low-cholesterol rabbit model of atherosclerosis using healthy rabbits as controls. Low inflation pressures were used to spare the abnormal but nonstenotic vessel wall.

## Materials and Methods

### Rabbit Model of Atherosclerosis

**Fig. 1:**
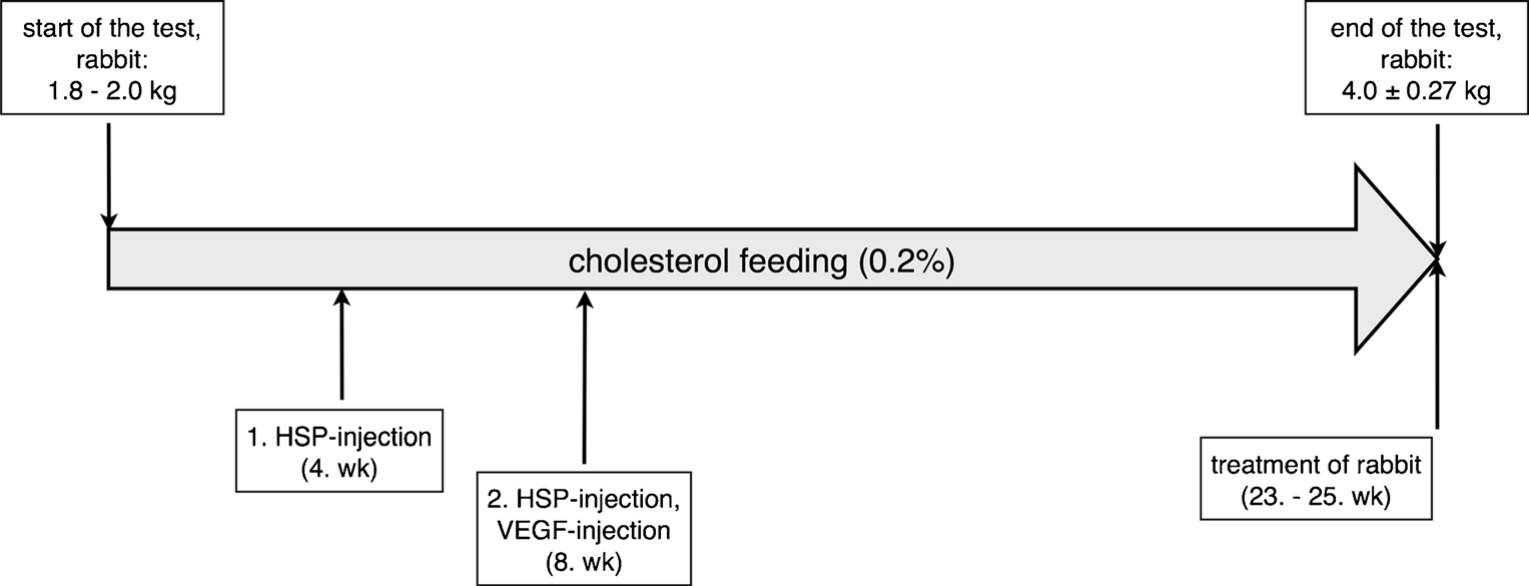
Time course of induction of atherosclerotic plaques in rabbits. The boxes above the *arrow* list the mean weights of the atherosclerotic rabbits at the beginning and the end of the experiment. Below the time *arrow*, the boxes list the treatments of the atherosclerotic rabbits by time point in weeks (wk) after the beginning of the cholesterol diet

**Fig. 2:**
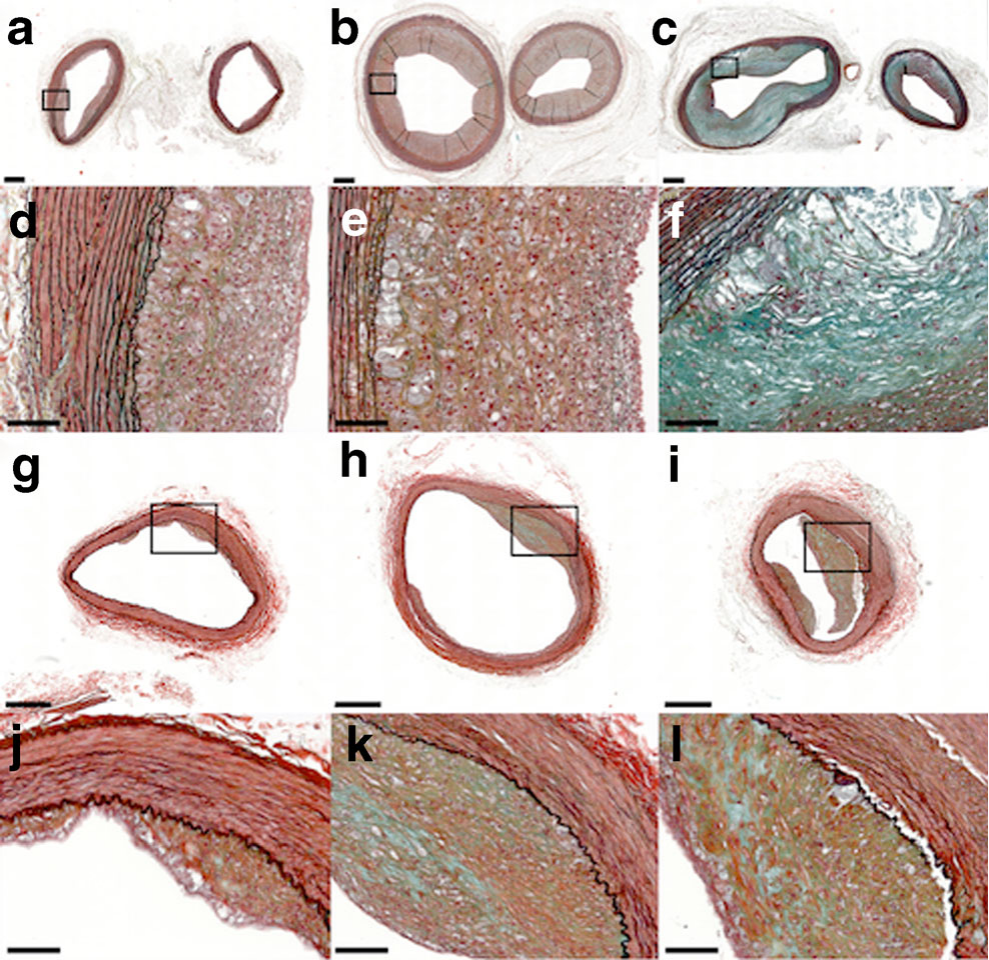
Representative histological sections of atherosclerotic lesions induced in the used rabbit model. Movat‘s pentachrome stain of mild to advanced atherosclerotic lesions in the brachiocephalic trunk (**a**-**c** left), left common carotid artery (**a**-**c** right), and iliac artery (**g**-**i**); *bar* =500 μm. The framed areas are magnified in (**d**-**f)** and (**j**-**l)**; *bar* =100 μm

The experiments were conducted in male New Zealand White Rabbits (Charles River Laboratories International, Inc., Sulzfeld, Germany). To induce atherosclerotic lesions the rabbits were fed a 0.2 % cholesterol diet (Altromin Spezialfutter GmbH, Lage, Germany) ad libitum for five to six months. Atherosclerotic lesion development was enhanced by two subcutaneous injections of heat shock protein (HSP) (HSP 65 kD from Mycobacterium bovis BCG, fragments 180–188, Sigma-Aldrich Chemie GmbH, Steinheim, Germany) [18–22] and a single intravenous (IV) injection of vascular endothelial growth factor (VEGF) (recombinant human VEGF, Sigma-Aldrich Chemie GmbH, Steinheim, Germany) [23, 24]. The first HSP (100 μg/animal in 1 mL PBS) injection was given after 4 weeks on the cholesterol diet. The second HSP injection (boost; 100 μg/animal in 1 mL PBS) and the VEGF injection (5 μg/animal in 0.5 mL aqua ad iniectabilia) were given after 8 weeks on the cholesterol diet (Fig. 1). Representative histological sections of atherosclerotic lesions induced in the rabbits are shown in Fig. 2.

### Paclitaxel-Coated Balloons

All balloons used in this study for coating with paclitaxel were commercially available high pressure balloons from Invatec (Invatec Technology Center GmbH, Frauenfeld, Switzerland) or Creganna (Creganna Medical, Galway, Ireland). The balloon membranes are made of nylon. The following balloon sizes were used: 3.5–20, 4.0–10, 4.0–20, 5.0–20, and 6.0–20 mm. All balloons were coated with 3.5 μg paclitaxel /mm^2^ balloon surface by InnoRa GmbH (Berlin, Germany), applying the Paccocath™ composition with the X-ray contrast medium iopromide as excipient. The balloons had a nominal pressure of 7 atm and a rated burst pressure of approximately 15 atm.

### Experimental Design

The rabbits were treated with PCB 23 to 25 weeks after the initiation of the cholesterol diet. Treatment was performed in 10 rabbits with atherosclerotic lesions induced as described above and in 13 healthy control rabbits. A total of 61 arterial segments (from 10 rabbits with atherosclerotic lesions and 13 healthy control rabbits; Table 1) were treated. At the time of intervention, mean body weight was 3.6 ± 0.2 kg in the 13 control rabbits and 4.0 ± 0.3 kg in the 10 atherosclerotic rabbits.

**Table 1:** Percentage of paclitaxel released from balloons at different inflation pressures

Inflation pressure [atm]	n	Healthy arteries [% of dose]	n	Atherosclerotic arteries [% of dose]
1	13	89.1 (88.2–93.1)	6	90.2 (78.2–95.9)
2	13	93.0 (90.0–95.1)	10	90.5 (82.2–94.3)
6	13	90.7 (71.5–92.7)	6	88.2 (71.5–92.3)
p*		0.0649		0.785

Data are given as median and IQR. * *p*-value of Kruskal-Wallis test (1 vs. 2 vs. 6 atm)

Anesthesia was induced with subcutaneous ketamine hydrochloride (Ketamin 10 %, WDT, Garbsen, Germany) at a dose of 35 mg/kg and medetomidine hydrochloride (Domitor®, Orion Pharma, Espoo, Finland) at a dose of 0.25 mg/kg. Intravenous maintenance doses were administered during the interventions as required. The rabbits were supplied with oxygen while being anesthetized. Throughout the interventions, ECGs were recorded and oxygen saturation was monitored.

**Fig. 3:**
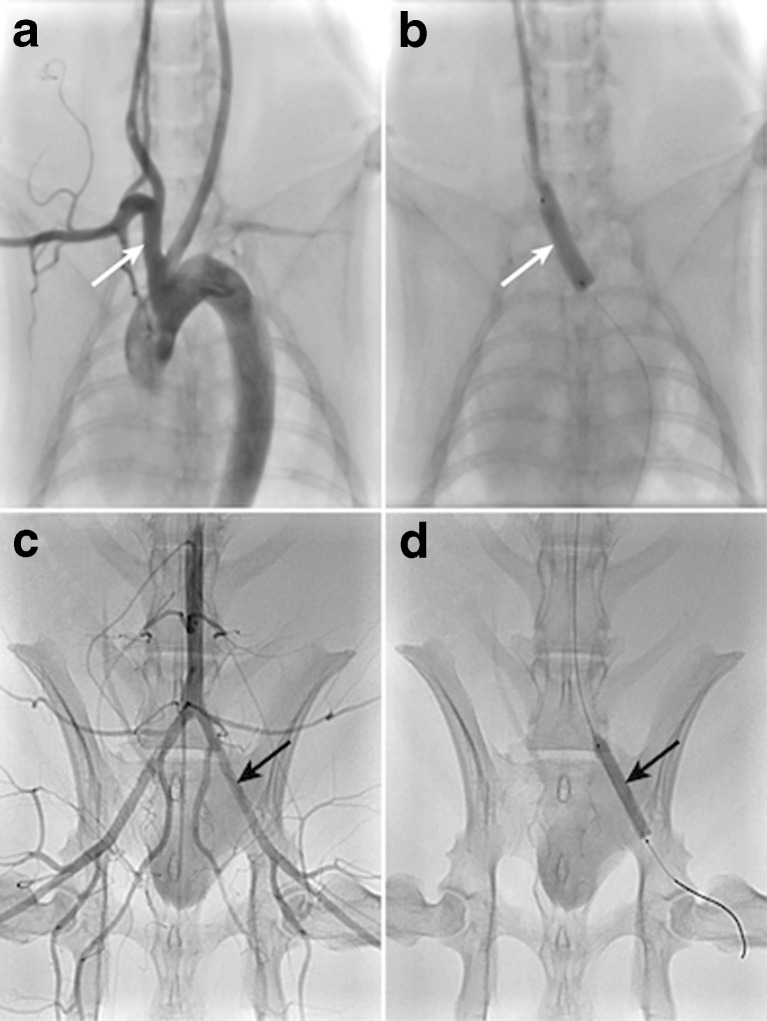
Overview of arterial sites selected for balloon inflation. Pre-treatment angiograms of the brachiocephalic trunk (**a**, *white arrow*) and iliac arteries (**c**, *black arrow* indicating left iliac artery); inflated balloons in these arteries are depicted in (**b**) and (**d**)

**Fig. 4:**
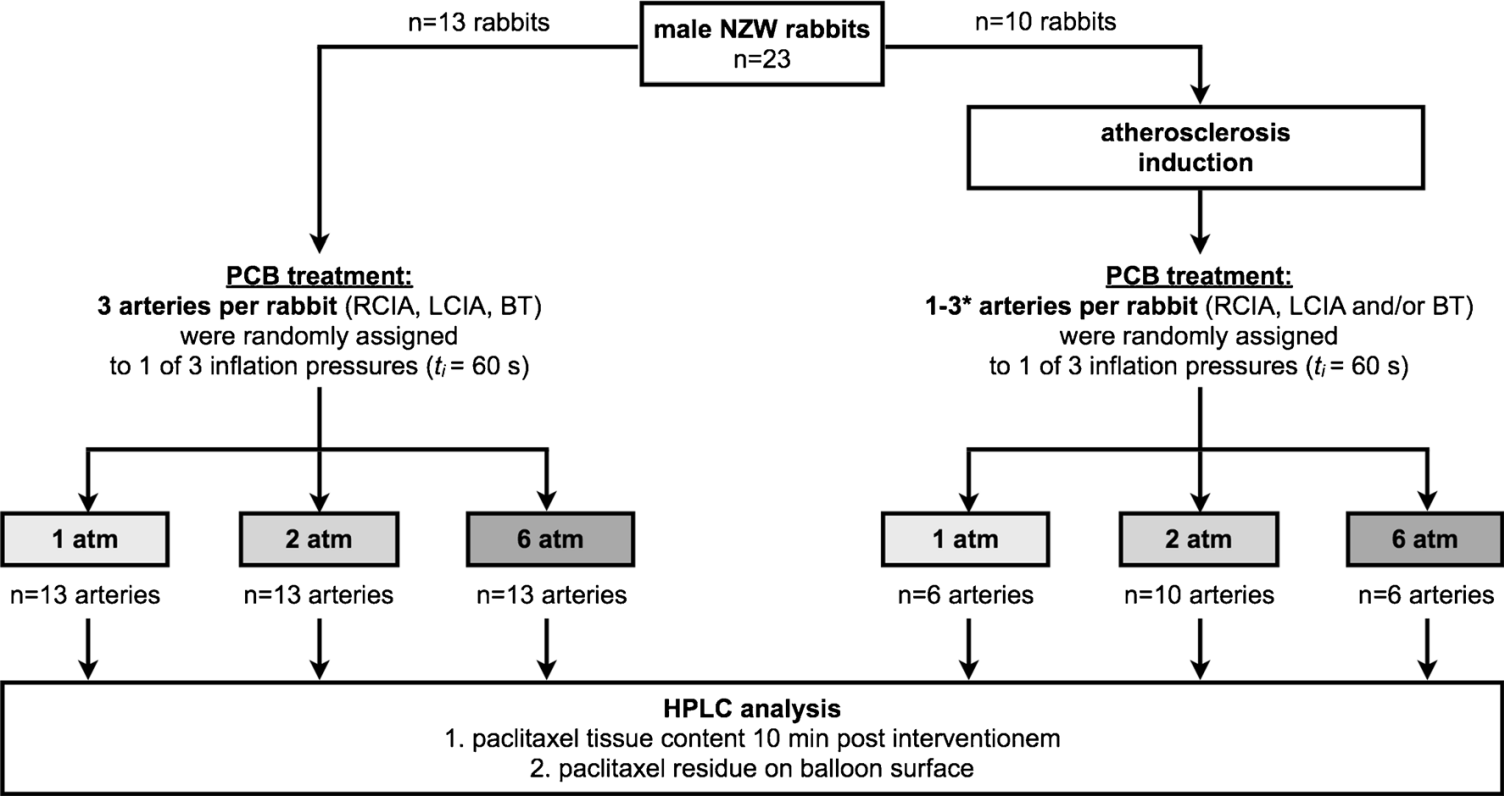
Experimental design and study groups. Drug transfer from paclitaxel coated balloons (PCB) was investigated in healthy (left side of diagram) and atherosclerotic arteries (right side of diagram) of New Zealand White (NZW) rabbits at 3 different inflation pressures; arteries selected for balloon inflation were the right and left common iliac artery (RCIA, LCIA) and the brachiocephalic trunk (BT). High-performance liquid chromatography (HPLC) was used to determine paclitaxel tissue content and paclitaxel remaining on the balloon surface. *t*_*i*_, inflation time. * Number of PCB treated arteries varies between 1 and 3 due to concurrent testing of additional substances in atherosclerotic rabbits (data not shown); target sample size for atherosclerotic rabbits was *n* = 6 arteries per subgroup

Arterial access was obtained via the right common carotid artery. Immediately before the interventions, all rabbits received 500 IU heparin through the arterial sheath (Heparin-Natrium-25,000-ratiopharm®, ratiopharm GmbH, Ulm, Germany). Baseline angiography was performed using a Siemens AXIOM Artis zee interventional angiography system following a iopromide bolus injection (Ultravist 370®, Bayer Health Care, Berlin, Germany). Segments for treatment were selected in the left and right iliac arteries and in the brachiocephalic trunk (BT) (Fig. 3). In the atherosclerotic and healthy animals, PCBs were expanded in a total of 22 and 39 arteries, respectively (Fig. 4). The inflation pressures used (1, 2, or 6 atm) were assigned randomly. The balloon diameters exceeded the reference diameters of the target arteries by 0.5–1 mm as estimated by angiography (mean overstretch ratio 1.5 ± 0.3). Estimation of the reference diameters was performed by quantitative measurements using the software included in the angiography system. Oversized balloons were chosen to ensure full circumferential wall contact in spite of low pressures. Balloon inflation time was 60s.

Ten minutes after the end of the intervention the rabbits were euthanized by IV pentobarbital injection (Narcoren®, Merial GmbH, Hallbergmoos, Germany), and the treated arterial segments were removed for measurement of paclitaxel content and concentrations in the vessel walls. In addition, we determined the amount of paclitaxel remaining on the balloons after use.

All experiments were performed in the Institute of Medical Technology & Research (Rottmersleben, Germany) and the Department of Radiology, Charité - Universitätsmedizin Berlin. This study was conducted in accordance with the requirements and guidelines of the EU legislation directive 2010/63/EU and the German Animal Protection Act. The experiments were approved by the local animal protection committee of the Sachsen-Anhalt government and of the LAGeSo Berlin, Germany.

### Paclitaxel Analysis

Paclitaxel was determined by high-performance liquid chromatography (HPLC) with ultraviolet detection. Paclitaxel remaining on the balloons after use was analyzed after extraction with ethanol. For extraction of arteries, a defined volume of ethanol was added, and the samples were treated with ultrasound for 30 min at room temperature and then centrifuged.

Paclitaxel extracted from balloons was analyzed on a Waters Symmetry C18 column (5-μm, 250 mm × 4.6 mm) using 55:45 (*v*/v) acetonitrile/0.005 M potassium phosphate buffer (pH 3.5) as mobile phase for balloon samples and 45:55 (*v*/v) acetonitrile/potassium phosphate for tissue samples (flow rate: 1 mL/min). Retention time of paclitaxel was ≈ 9 min in balloon samples and ≈ 22 min in tissue samples; detection was performed at 230 nm.

### Statistical Analysis

GraphPad Prism (Version 5.0a for Mac OS X; GraphPad Software, Inc.) was used for statistical analysis. Quantitative data are presented in box-and-whisker plots. Differences between two independent parameters were assessed for significance using the Mann-Whitney U-test. For comparison of more than two independent nonparametric samples, the Kruskal-Wallis test was used. Data are given as median and interquartile range (IQR) or mean ± standard deviation. The probability of error is given as p. A two-sided *p*-value of <0.05 is considered to indicate statistical significance.

## Results

### Treatment with PCB

All animals were successfully treated by balloon inflation. During the balloon interventions, only a few rabbits showed minimal variations in heart rate or short mild drops in oxygen saturation.

### Paclitaxel Release from Balloons

The median amount of paclitaxel remaining on the balloons (*n* = 61) after use (= proportion of dose not released during the procedure) was 0.29 μg/mm^2^ (IQR 0.21–0.41 μg/mm^2^) of balloon surface area. This corresponds to a median paclitaxel release during the procedures of 90.3 % (IQR 86.0–93.7 %). The median paclitaxel release from balloons used in healthy controls (*n* = 39 arteries) was 91.1 % (IQR 87.7–93.1 %) versus 89.8 % (IQR 78.2–94.3 %) in rabbits with atherosclerotic lesions (*n* = 22 arteries; *p* = 0.7582). Furthermore, we compared paclitaxel release between the three arterial segments that were treated: BT (*n* = 17), right iliac artery (*n* = 22), and left iliac artery (*n* = 22). Median paclitaxel release was 91.4 % (IQR 88.9–92.4 %), 89.9 % (IQR 71.1–93.7 %), and 90.3 % (IQR 83.1–94.7 %), respectively (*p* = 0.8689). Neither in healthy arteries (*p* = 0.0649) nor in atherosclerotic arteries (*p* = 0.785) was the percentage paclitaxel release significantly dependent on the inflation pressure applied (Table 1).

### Paclitaxel in Tissue

In healthy arterial segments, the median paclitaxel tissue content was 21.16 μg (IQR 11.18–33.04 μg), which corresponds to a median paclitaxel tissue concentration of 829.3 μg/g (IQR 636.5–1487 μg/g) and 1.90 % (IQR 1.10–2.79 %) of the paclitaxel dose.

In atherosclerotic arterial segments, median tissue recovery was 8.83 μg (IQR 2.52–14.67 μg), corresponding to a median paclitaxel tissue concentration of 375.7 μg/g (IQR 169.8–771.6 μg/g) and 1.00 % (IQR 0.38–1.86 %) of the paclitaxel dose. The paclitaxel tissue concentration differed significantly between healthy and atherosclerotic arteries (*p* = 0.0002) with concentrations being lower in atherosclerotic arterial segments.

Data of paclitaxel tissue recovery at the different inflation pressures are summarized in Table 2. No statistically significant relationship was observed between paclitaxel concentrations in healthy arteries and the inflation pressures applied (*p* = 0.5382), whereas in atherosclerotic arteries, an increase in balloon inflation pressure resulted in higher paclitaxel tissue concentrations (*p* = 0.0106, Table 2). At a pressure of 6 atm, tissue concentrations were comparable to those achieved in healthy controls.

**Table 2:** Paclitaxel recovery in arterial tissue

	*p*_*i*_			Paclitaxel in tissue	
	[atm]	n	[μg]	[% of dose]	[μg/g]
	1	13	18.64 (14.29–31.74)	1.68 (1.16–2.76)	792.7 (520.2–1211)
Healthy	2	13	22.26 (18.37–31.55)	2.04 (1.69–2.42)	823.6 (549.6–2059)
arteries	6	13	20.83 (8.78–52.94)	2.07 (0.88–5.59)	875.3 (756.0–1493)
	p*		0.7906	0.6419	0.5382
	1	6	2.51 (1.69–6.57)^††^	0.40 (0.26–0.71)^††^	212.4 (136.4–296.1)^††^
Atherosclerotic	2	10	9.17 (3.88–11.22)^††^	1.12 (0.33–1.77)^††^	429.0 (132.1–616.6)^†^
arteries	6	6	16.22 (13.04–27.07)	2.65 (1.30–4.29)	926.8 (644.9–1114)
	p*		0.0038	0.0081	0.0106

Data are given as median and (IQR)

*p*_*i*_, inflation pressure

*p-value of Kruskal-Wallis test (1 vs. 2 vs. 6 atm)

^†^ *p* < 0.05

^††^ *p* < 0.01 in Mann-Whitney test (atherosclerotic vs. healthy arteries at the same inflation pressure)

Paclitaxel tissue concentrations in healthy and atherosclerotic arteries did not differ significantly between the BT and the right and left iliac arteries (*p* = 0.9783 and *p* = 0.4777). Median concentrations in healthy arteries were 927.6 μg/g (IQR 617.4–1424 μg/g, *n* = 13) in the BT, 829.3 μg/g (IQR 520.2–1542 μg/g, *n* = 13) in the right iliac artery, and 792.7 μg/g (IQR 704.6–1428 μg/g, *n* = 13) in the left iliac artery. In atherosclerotic arteries, median concentrations were 243.0 μg/g (IQR 57.4–1049 μg/g, *n* = 4), 342.0 μg/g (IQR 166.3–650.3 μg/g, *n* = 9), and 472.1 μg/g (IQR 265.6–955.3 μg/g, *n* = 9), respectively.

## Discussion

The efficacy and safety of PCB was first demonstrated in clinical settings by Scheller et al. (2006) and Tepe et al. (2008) [25, 26] and has recently been confirmed by a large randomized multicenter study [27]. In this study (IN.PACT SFA) PCB have been tested for the treatment of symptomatic superficial femoral and proximal popliteal artery disease in comparison to percutaneous transluminal angioplasty (PTA) with uncoated balloon catheters. PCB resulted in persistently lower rates of target lesion revascularization compared with uncoated balloons (2.4 % vs. 20.6 %) at 12 months [27]. Moreover, the efficacy and safety of PCB (with or without stent in the treated segment) has also been demonstrated in clinical studies for a variety of indications such as in-stent restenosis [13, 28–31], de novo stenosis [32–34], stenosis of small vessels [35, 36] and bifurcation stenosis [37, 38].

In the clinical cases (PTA/PTCA), high pressures of ≥6 atm are usually employed to dilate stenotic arteries. Most animal studies of PCB were also performed using high inflation pressures (6–15 atm) [14, 15, 39–42] in the established model of coronary artery overstretch and stenting in pigs [22, 43, 44]. To the best of our knowledge, there is only one study in which PCB were inflated using low pressure. Cremers et al. [45] used the model of restenosis in pigs, comparing the angiographic parameter of late lumen loss 28 days after interventions. They implanted bare metal stents in the coronary arteries, followed by repeat dilatation with PCB using inflation pressures of 2 and 12 atm. The investigators identified no significant differences in effectiveness between the two pressures used, but they did not measure paclitaxel tissue concentrations in the arterial walls. Therefore, we cannot compare the amount of paclitaxel recovered in the arterial wall following balloon inflation at 2 atm in our experiments in peripheral arteries of rabbits with the effective amount of paclitaxel transfer in the study of Cremers et al. [45]. In our experiments, only atherosclerotic arteries showed a statistically significant dependence of the paclitaxel tissue concentration on the balloon inflation pressure used. In healthy vessels, there was only a trend toward higher paclitaxel transfer with increasing pressures.

In the atherosclerotic arterial segments of the rabbits analyzed in our study, mean recovery of paclitaxel for all treatments taken together was 1.00 % of the initial dose on the balloons. This corresponds to a median paclitaxel tissue concentration of 375 μg/g and is 2.2 times lower than the concentration in healthy arteries in the same species. Nevertheless, this concentration is still clearly within the effective range of paclitaxel compared with the tissue concentration known to inhibit neointimal proliferation in pigs [15, 40, 42]. In a study in young domestic pigs, Speck et al. [42] recovered 5.5 ± 4.7 % of the initial paclitaxel dose in the arterial wall 10 to 30 min after inflation of PCB (3 μg/mm^2^) at high pressure (12.1 ± 1.4 atm). This amount corresponded to a paclitaxel tissue concentration of 133 ± 114 μg/g. In a second study, Speck et al. [42] investigated the amount of paclitaxel in the vessel walls of adult minipigs using the same procedures as in the first study. In the vessel walls of the minipigs, they recovered 21.1 ± 7.6 % of the initial paclitaxel dose (534 ± 285 μg/g) 10 to 30 min after balloon inflation at a pressure of 12.2 ± 1.3 atm. However, in the second study, the experiments were performed using PCB with premounted stents. The rabbit arteries investigated in our experiments are much thinner than porcine arteries. The mean paclitaxel concentration we recovered in healthy rabbit arteries was 1141 ± 809 μg/g of tissue (median: 829.3 μg/g), which is 8.6 times higher than the concentration in young pigs and 2.1 times higher than the concentration in adult minipigs in the studies of Speck et al. The mean tissue concentration of 502 ± 385 μg/g (median: 375.7 μg/g) in atherosclerotic arteries was also still above that in domestic pigs [42] and the values reported by Buszman et al. [46], which correspond to tissue concentrations of approximately 153 ± 155 ng/mg. A recently published pharmakokinetic study by Fernández-Parra et al. (2015) [47] reported paclitaxel tissue concentrations of 632 ± 126 ng/mg (mean ± SEM) in atherosclerotic iliac arteries of rabbits. However, the authors did not mention the inflation pressure applied in their study. The calculated paclitaxel tissue concentration (1 h after angioplasty) was similar to our data. Contrary to our experiments Fernández-Parra et al. determined a paclitaxel tissue concentration of 169 ± 83 ng/mg in the arteries of the control animals. This lower concentration might be explained by the use of a different histologic vessel preparation (washing of the arteries after dissection).

Treatment with PCB may be the final step after high-pressure predilatation (8–15 atm) of stenotic vessel segments and aims at restoring the original vessel lumen and eliminating the flow obstacle. The initial interventional maneuvers lead to vessel injury, excision or compression and rupture of plaques, and distention of the arterial wall [48–50]. Once a sufficiently large luminal diameter has been restored, use of a PCB may smoothen the luminal surface of the vessel and inhibit neointimal proliferation, and even facilitate plaque regression [51–53]. Furthermore, it is assumed that unstable plaques often have no clinical relevance and may even go unnoticed in coronary angiography [54]. When a PCB is used in the presence of unstable plaques, high-pressure inflation might be harmful. Also, when the original lumen diameter is restored at the site of a significant atherosclerotic lesion, the arterial segment may be susceptible to rupture. In some challenging cases such as unstable plaques or after prior treatment with an atherectomy catheter, it could therefore be advisable to use lower pressures when the aim of treatment is to stabilize lesions, slow further plaque progression and lumen loss, or stimulate metabolic degradation of atherosclerotic material after its compression to the vessel wall. Preventive percutaneous transluminal (coronary) angioplasty (PTCA/PTA) for the treatment of unstable atherosclerotic lesions might be a further indication for the use PCB. Moreover, PCB treatment at low pressure may smoothen luminal surfaces of arteries, which might be beneficial for edge areas after stent implantation.

Interestingly, our experiments confirm that, even with low inflation pressures and low transfer efficacy, high tissue concentrations of paclitaxel are achieved in the delicate arteries of rabbits. Therefore, based on the results of our rabbit experiments, we may assume that a balloon inflation pressure of 2 atm is more than sufficient to deliver a potentially effective paclitaxel concentration to the vessel wall.

### Study Limitations

Atherosclerotic lesions in rabbits have some resemblance with such lesions in humans. However, no animal model is currently available that simulates all aspects of human atherosclerosis development and the heterogeneity of factors involved, ranging from cellular components to inflammatory reactions or calcifications.

Since the tissue mass and vessel wall thickness of rabbit arteries is markedly lower than that of pig arteries, our results cannot be readily compared with published results obtained in pigs. Further studies have to show whether paclitaxel is effective in treating atherosclerotic lesions in rabbits and ultimately in patients.

## Conclusion

In the present study we investigated paclitaxel transfer from PCB into atherosclerotic vessel walls and compared the results with healthy controls. Specifically, we investigated whether paclitaxel transfer is affected by the applied balloon inflation pressures (1, 2, and 6 atm) and the selected artery. Our results are as follows:

1. Atherosclerotic lesions impede the transfer of paclitaxel into arterial walls.
2. Higher inflation pressures increase paclitaxel transfer in atherosclerotic arteries; in healthy arteries the effect of inflation pressure is less obvious or absent.
3. Tissue concentrations achieved with an inflation pressure of 2 atm are potentially effective in this atherosclerotic model.
